# Two case reports of breast cancer combined with synchronous primary intrahepatic cholangiocarcinoma/mixed liver cancer

**DOI:** 10.1097/MD.0000000000040653

**Published:** 2024-11-29

**Authors:** Miduo Tan, Liu Luo, Taoli Wang, Zhiyong Zhang, Yuqin Wei, Chenyan Long

**Affiliations:** a Department of Breast Surgery, The Affiliated Zhuzhou Hospital of Xiang Ya School of Medicine Central South University, Zhuzhou, China; b Department of Anesthesiology, The Affiliated Zhuzhou Hospital of Xiang Ya School of Medicine Central South University, Zhuzhou, China; c Department of Pathology, The Affiliated Zhuzhou Hospital of Xiang Ya School of Medicine Central South University, Zhuzhou, China; d Department of General Surgery, The Affiliated Zhuzhou Hospital of Xiang Ya School of Medicine Central South University, Zhuzhou, China; e Graduate School of Oncology, Guangxi Medical University, Nanning, China; f Division of Colorectal and Anal Surgery, Department of Gastrointestinal Surgery, Guangxi Medical University Cancer Hospital, Nanning, China.

**Keywords:** breast cancer, case report, intrahepatic cholangiocarcinoma, mixed liver cancer, multiple primary malignant tumors

## Abstract

**Rationale:**

This case report discusses multiple primary malignant tumors, which refer to the occurrence of 2 or more different histological types of malignant tumors simultaneously or successively in the same individual.

**Patient concerns:**

We present 2 female patients who were admitted to the hospital due to a “left breast mass” and were found to have multiple solid masses in the liver upon imaging.

**Diagnoses:**

Postoperative pathology revealed that one patient had breast invasive ductal carcinoma was complicated with primary intrahepatic cholangiocarcinoma and mixed hepatocellular carcinoma with intrahepatic cholangiocarcinoma.

**Interventions:**

Both patients underwent extensive resection of the lesion.

**Outcomes:**

Regular postoperative checkups and follow-ups have been conducted, and both patient’s current conditions are stable.

**Lessons:**

The treatment approach adopted in this case report may serve as a favorable reference for the management of similar cases. However, further extensive biological studies are still needed to investigate the biological mechanisms of multiple primary malignant tumors and to discover specific therapeutic approaches to achieve more clinical benefits for patients.

## 1. Introduction

Multiple primary malignant neoplasms (MPMNs) refer to the simultaneous or sequential occurrence of 2 or more primary malignant tumors in 1 or more organs of the same host. According to the discovery time interval of 6 months, it is divided into synchronous and metachronous. Warren^[1]^ proposed the diagnostic criteria for MPMNs in 1932: (1) histologically malignant; (2) each tumor has a unique pathological morphology; (3) different parts, the 2 are not continuous; and (4) exclusion of transfer. At present, reports on MPMNs mainly focus on head and neck tumors, digestive system tumors and lung cancer, and there are few reports on female multiple primary breast cancer malignant tumors.^[2]^ This study reported 2 patients with left breast invasive ductal carcinoma combined with intrahepatic cholangiocarcinoma and mixed liver cancer confirmed by pathological examination, which belonged to MPMNs and simultaneity. There are few reports on multiple primary malignant tumors in female breast cancer or cholangiocarcinoma. To the best of our knowledge, very few cases of primary intrahepatic cholangiocarcinoma/mixed liver cancer have been documented in female breast cancer. There are currently 5 published reports of primary breast cancer combined with hepatocellular carcinoma, 4 of which occurred in male patients^[3–6]^ and only 1 in female patients.^[7]^ In detail, most of the 5 reported cases attributed the cause of breast cancer combined with synchronous primary liver cancer to hormonal imbalance, without further discussion of more possible etiologies and pathophysiological changes of MPMNs. However, in our case report, we further comprehensively discussed the potential etiology, pathogenesis, clinical manifestations, diagnosis and treatment of MPMNs on the basis of detailed case presentation.

## 2. Case presentation

**Figure 1.**
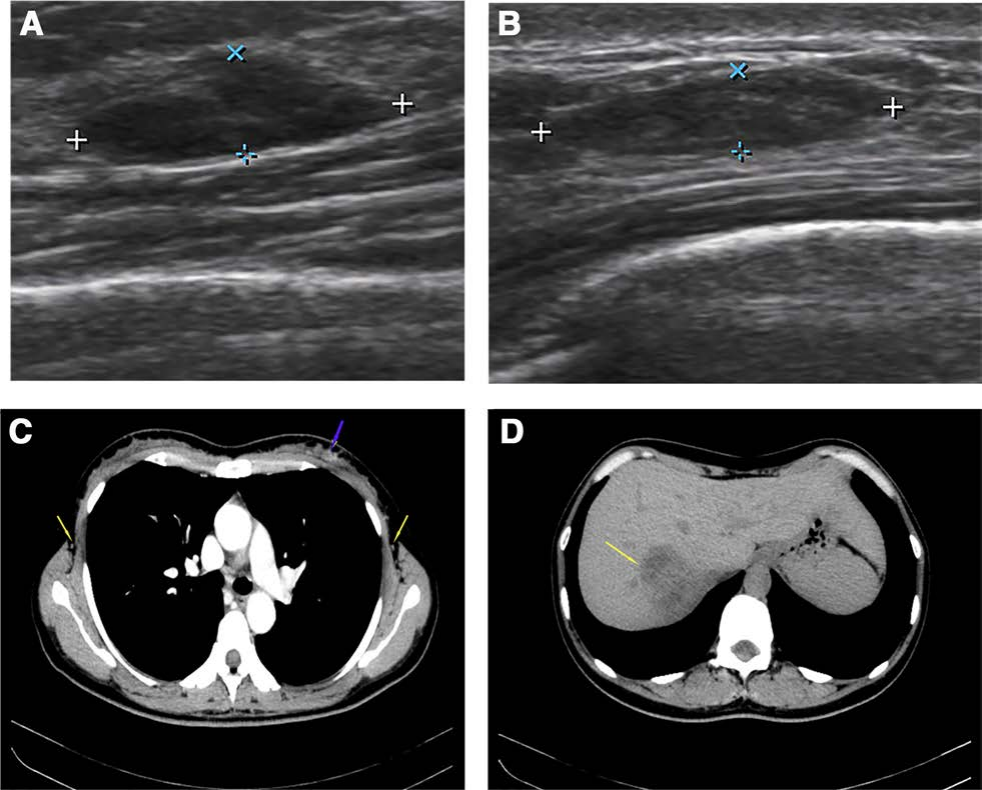
Case 1: Images of breast nodules and liver masses. The tissue structure of each layer of the bilateral breast was clear, the glandular tissue was relatively homogeneous, with a strong echo of fine honeycomb, and hypoechoic nodules were seen in the glands. The maximum left side was about 13 mm × 4 mm (12 o’clock direction, about 20 mm from the nipple). (A) The maximum right side was about 14 mm × 4 mm (7 o’clock direction, beside the areola). (B) Oval, parallel growth, complete edge, uniform internal echo, no punctate strong echo, the superficial fascia of the breast at the lesion was shallow and deep, and there was no obvious blood flow signal in the nodules. (C) Enhanced CT showed uneven density of the left breast, multiple punctate enhanced nodules in the left breast area with clear edges (purple arrows), and multiple small lymph nodes in the bilateral axilla (yellow arrows). (D) Plain CT scan showed multiple nodular, lumpy slightly low-density lesions (yellow arrows) in the right lobe of the liver, partially fused into sheets, with unclear boundaries, and a range of about 50 mm × 42 mm.

**Figure 2.**
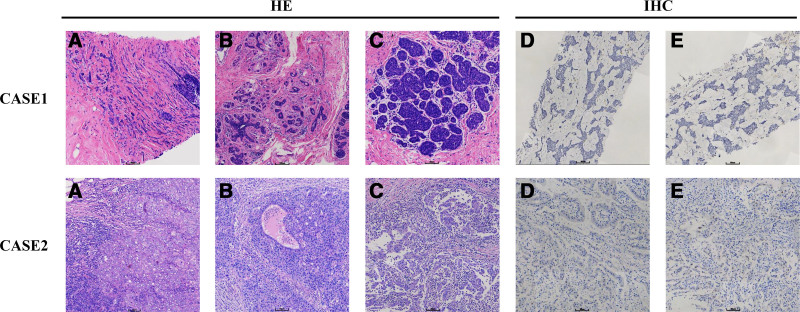
Cases 1 and 2: Pathological images of breast mass and liver mass. The morphology of breast mass and liver mass in these 2 patients were different under the microscope: Case 1: (A) The tumor tissue of breast tumor grew in cordlike or soldier shape, with small nucleus, no obvious nuclear division and interstitial collagen formation. (B) The breast mass showed a fibroadenoma of the breast. (C) The breast mass was a typical leaf in situ carcinoma. (D) The breast specific marker GATA3 in liver mass carcinoma was negative. (E) The breast specific marker Mammaglobin in liver mass carcinoma was negative. Case 2: (A) The tumor tissue of breast mass showed coarse bundle infiltrating growth, dispersed necrosis, nuclear vacuolation, visible nucleoli, obvious atypia, easy mitosis, interstitial fibrosis, and more infiltration of lymphocytes and plasma cells. (B) In the liver mass, the cancer cells in the liver cancer area were nested, with nuclear vacuoles, obvious atypia, mitosis, visible necrosis, and interstitial inflammatory cell infiltration (mainly neutrophils). (C) In the liver mass, the cancer cells in the cholangiocarcinoma area showed irregular adenoid or cribriform hyperplasia, nuclear vacuolation, nucleolar and interstitial neutrophil infiltration. The liver mass has 2 parts, poorly differentiated hepatocellular carcinoma and moderately differentiated cholangiocarcinoma. (D) The breast specific marker Mammaglobin in liver mass carcinoma was negative. (E) GCDFP-15 was negative in liver mass carcinoma.

*Case 1*: A 35-year-old female patient was admitted to the hospital with a left breast mass. On September 24, 2021, breast B-ultrasound showed multiple solid nodules in bilateral breasts (Fig. 1A and B), BI-RADS: 3; on September 26, 2021, minimally invasive rotary resection of double breast nodules was performed (Fig. 1C). Postoperative pathology showed invasive ductal carcinoma of the left breast, histologic grade I, with atypical lobular hyperplasia-transtentional lobular carcinoma in situ involving fibroadenoma. Immunohistochemistry: ER(+), PR(+), Ki-67(+), and Cerb-B2 breast cancer (BC)(+). The laboratory examination on October 12, 2021 is shown in Table 1; abdominal CT showed multiple nodules and masses in the right lobe of the liver, suggesting liver metastases (Fig. 1D); liver MRI: 1. Multiple nodules and masses in the right lobe of the liver, considering liver metastases; 2. Obvious enhancement of the right lobe of the liver, considering abnormal perfusion; 3. Multiple retroperitoneal lymph node enlargements were considered metastases. On November 9, 2021, left breast unilateral radical mastectomy with ipsilateral axillary sentinel lymph node biopsy was performed. The pathological results of the simple excision specimen of the left breast after minimally invasive rotary excision surgery and the left sentinel lymph node specimen showed residual classic lobular carcinoma in situ in a small amount of the submitted breast tissue, and no metastasis was found in the left sentinel lymph node. Immunohistochemistry: Ki-67(+), ER(+), PR(+), CK-Pan(+), Calponin(+), P63(+), E-cadherin(+), P120(+), and GATA3(+). As this patient has been diagnosed with hormone receptor-positive lobular carcinoma in situ and is in a premenopausal state, after surgery, goserelin (subcutaneous injection of anterior abdominal wall, 3.6 mg each time, 28 days/cycle) + letrozole (oral, 2.5 mg each time, 1 day/times) maintenance treatment was administered. On November 19, 2021, abdominal CT showed multiple nodules and masses in the liver, which were fed by the branches of the right hepatic artery. The right hepatic vein was involved, a tumor thrombus was formed in the lumen, and metastasis was considered. Liver MRI showed multiple nodules in the right lobe of the liver. Considering liver metastasis and right hepatic vein involvement, and multiple enlarged lymph nodes in the hilar and retroperitoneal abdominal aorta, metastasis may occur. On December 6, 2021, laparoscopic liver mass resection was performed under general anesthesia. The postoperative pathology and immunohistochemistry showed that the liver mass was primary intrahepatic cholangiocarcinoma (Fig. 2). Moreover, it is worth mentioning that the publication of the BILCAP 5-year OS results has demonstrated that capecitabine remains the standard adjuvant treatment for biliary tract cancer. Since this patient does not have indications for postoperative adjuvant radiotherapy, treatment with capecitabine (1250 mg/m^2^ twice daily, days 1–14, repeated every 3 weeks for a total of 8 cycles) has been administered. Regular postoperative follow-up. On September 30, 2022, a review of color Doppler ultrasound and contrast-enhanced ultrasound of the liver revealed multiple solid nodules of the liver to consider intrahepatic cholangiocarcinoma recurrence. Envafolimab was administered as immunotherapy (Envafolimab Injection, Envida) after thorough communication with the patient. Specifically, the patient received subcutaneous injections of 400 mg Envafolimab every 3 weeks. On December 18, 2022, abdominal MRI showed multiple nodules and masses of varying sizes in the liver parenchyma. The number of nodules and masses increased and some of them increased. The right hepatic vein may be invaded. On January 11, 2023, carbohydrate antigen (CA19-9) was found to be 98.69 U/mL. On the same day, the patient underwent a local anesthetic liver artery embolization procedure. On March 6, 2023, liver MRI showed multiple nodules and masses of varying sizes in the remaining liver parenchyma were reduced and shrunk compared with the previous. Continue to follow-up the patient regularly.

**Table 1:** Changes of related laboratory examination indexes during case1 diagnosis and treatment.

Parameters	Reference values	Preoperative (2021.10.11)	Postoperative (2021.12.07)	Recheck		Postoperative TACE (2023.01.14)
				(2022.01.04)	(2023.01.11)	
White blood cell count (10^9^/L)	3.5–9.5	25.2	22.1	7.7	6.1	8.9
Red-cell count (10^12^/L)	3.80–5.10	4.31	4.05	3.91	3.72	3.58
Hemoglobin (g/L)	115–150	131	124	111	117	116
Thrombocyte (10^9^/L)	125–350	243	190	256	162	91
Albumin (g/L)	40.0–55.0	30.9	41.9	42.4	34.8	36.3
Alanine aminotransferase (U/L)	7.0–40.0	686.0	608.3	25.5	9.5	83.4
Aspartate aminotransferase (U/L)	13.0–35.0	867.2	582.3	26.7	19.7	130
Total bilirubin (µmol/L)	0.0–25.0	20.0	54.9	8.7	14.3	39.7
Direct bilirubin (µmol/L)	0.0–6.8	7.1	20.6	2.3	5.2	15.1
Abnormal prothrombin (PIVKA II) (mIU/mL)	11.12–32.01	26.90	/	/	/	/
Urea (UREA) (mmol/L)	2.60–7.50	/	3.56	/	3.64	3.94
Creatinine (CREA) (µmol/L)	41.0–111.0	/	67.40	/	53.00	31.8
Blood sugar (GLU) (mmol/L)	3.90–6.10	/	/	/	4.54	/
Hepatitis B virus surface antibody (mIU/mL)	0–10	>1000.00	/	/	/	/
Hepatitis B core antibody (S/CO)	0.00–0.90	3.74	/	/	/	/
Alpha-fetoprotein (AFP) (ng/mL)	0.00–7.00	1.77	/	/	/	/
Carcinoembryonic antigen (CEA) (ng/mL)	<5.00	0.83	/	/	0.89	/
Carbohydrate antigens (CA125) (U/mL)	<35.00	12.20	/	/	/	/
Carbohydrate antigens (CA15-3) (U/mL)	<15.00	11.20	/	/	/	/
Carbohydrate antigens (CA 19-9) (U/mL)	<37.00	23.61	/	/	87.69	/

**Figure 3.**
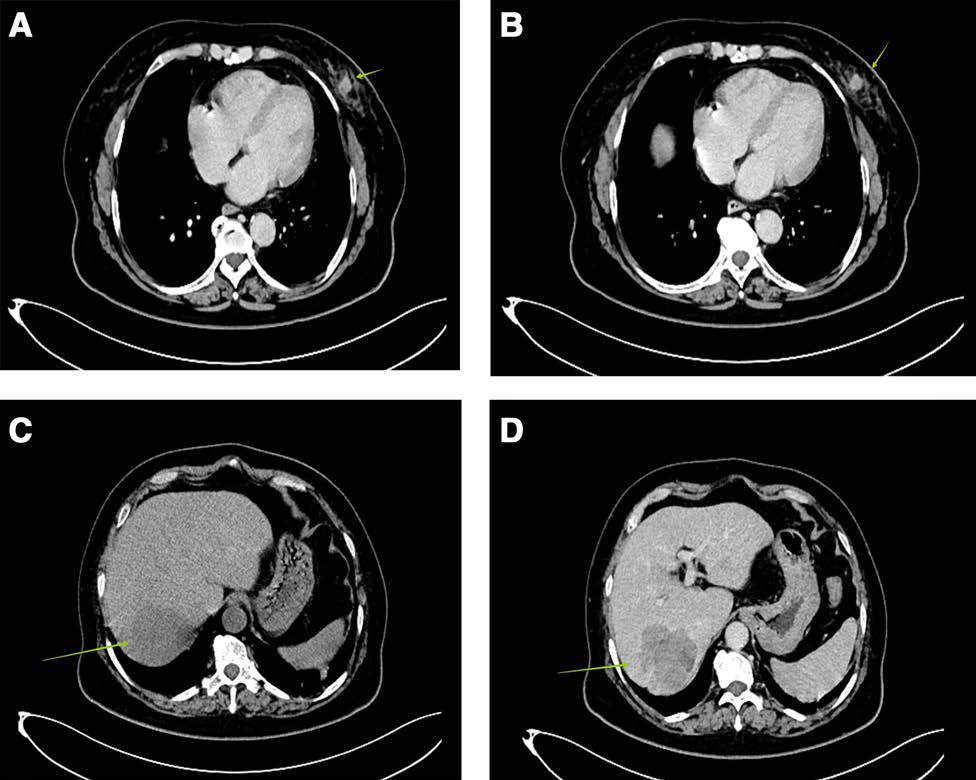
Case 2: Cross-sectional CT images of breast and liver masses. (A) Enhanced CT portal phase showed an irregular mass (short green arrow) in the left breast area, with slightly uniform density, clear boundary, and obvious fibrous cords around. (B) Enhanced CT portal phase showed a round-like mass (short green arrow) in the left breast area, with peripheral ring enhancement, clear boundary, and a few fibrous cords around. (C) Plain CT scan showed low-density irregular masses in S6 and 7 segments of the right posterior lobe of the liver with clear boundaries (long green arrow). (D) Enhanced CT portal phase showed irregular enhancement of S6 and 7 segments of the right lobe of the liver (long green arrow).

**Figure 4.**
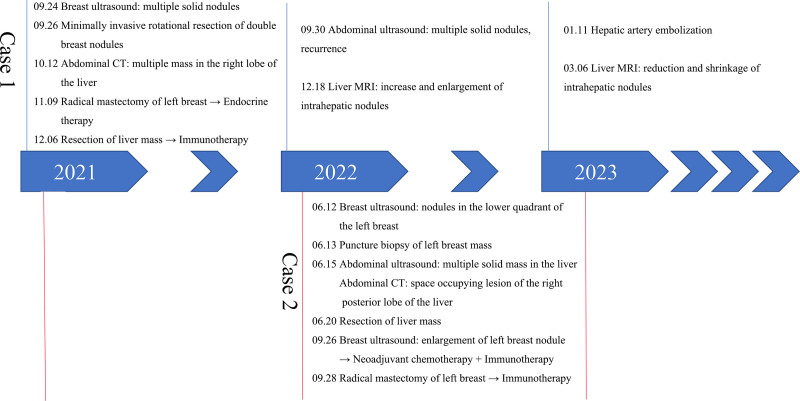
Axis diagram of diagnosis and treatment process. The main diagnosis and treatment process of the disease development of 2 patients.

*Case 2*: A 62-year-old woman was admitted to the hospital on June 12, 2022, with a “1-day history of a left breast mass.” On physical examination, a round mass, approximately 1.5 cm × 1.5 cm, was palpated approximately 2 cm from the areola at 5 o’clock in the lower outer quadrant of the left breast. It was of medium texture, ill-defined, smooth, poorly mobile, nontender, and did not produce nipple discharge when the mass was pressed. Multiple lymph nodes were touched in the left axilla, and the larger one was a round mass about 1.5 cm × 1.5 cm with medium texture, clear border, smooth surface, good mobility, and no tenderness. And ultrasonography showed a nodule in the lower quadrant of the left breast, BI-RADS: 4B. On June 13, 2022, ultrasound-guided needle biopsy of the left breast mass was performed (Fig. 3A and B). Pathological examination showed invasive ductal carcinoma with necrosis, histological grade III. No metastatic carcinoma was found in the left axillary lymph node tissue. Immunohistochemistry: Cerb-B2 BC(+), CK5/6(+), P63(+), P120(+), E-cadherin(+), AR(+), EGFR(+), Ki-67(+). Laboratory examination is shown in Table 2; liver B-ultrasound suggested multiple solid masses in the liver; liver CT showed space-occupying lesions in the right posterior lobe of the liver: metastatic tumor (Fig. 3C and D)? Due to the higher malignancy of the liver lesion compared to the breast lesion, in accordance with the patient’s wishes, radical liver cancer surgery was performed first, followed by a period of recovery before proceeding with breast cancer surgery. On June 20, 2022, laparoscopic exploration + conversion to open resection of S6 and 7 tumors of the right liver was performed under general anesthesia. Postoperative pathology showed primary mixed hepatocellular carcinoma (poorly differentiated) with intrahepatic cholangiocarcinoma (moderately differentiated). Immunohistochemistry: AFP(+), Hepatocyte(+), Ki-67(+), CK7(+), CK20(+), GS(+), Syn(+), CD34(+), CK19(+), Ki-67(+), and CK7(+) (Fig. 2). After the liver cancer surgery, no specific additional treatment was administered, and regular follow-up exams were recommended. Meanwhile, due to the breast cancer being in its early stage, the TC chemotherapy regimen was administered according to the guidelines, which included docetaxel (140 mg), cyclophosphamide (1.0 mg), and trastuzumab (440 mg) for a total of 4 cycles. Concurrently, a year of targeted therapy targeting the HER-2 gene with trastuzumab (330 mg for 13 cycles) was also administered. On September 26, 2022, a breast ultrasound examination revealed an enlargement of the left breast nodule compared to previous exams. Subsequently, on September 28, 2022, under general anesthesia, the patient underwent a left breast resection with sentinel lymph node biopsy on the same side. The postoperative pathology was invasive ductal carcinoma (T1N0M0 stage I). Immunohistochemistry: Ki-67(+), Cerb-B2 BC(+). The tumor did not progress. The diagnostic and treatment processes, as well as the differential indicators of medical history between case 1 and case 2, have been summarized in Figure 4 and Table 3, respectively.

**Table 2:** Changes of related laboratory examination indexes during the second diagnosis and treatment of case 2.

Parameters	Reference values	Preoperative (2022.06.12)	Postoperative (2022.06.24)	Recheck (2022.09.26)
White blood cell count (10^9^/L)	3.5–9.5	6.71		5.33
Red-cell count (10^12^/L)	3.80–5.10	4.52		4.21
Hemoglobin (g/L)	115–150	136.00		125.00
Thrombocyte (10^9^/L)	125–350	176.00		186.00
Alanine aminotransferase (ALT) (U/L)	7.0–40.0	29.00		38.00
Aspartate aminotransferase (AST) (U/L)	13.0–35.0	59.00	81.00	66.00
Total bilirubin (µmol/L)	0.0–25.0	30.20	15.90	15.60
Direct bilirubin (µmol/L)	0.0–6.8	9.30	7.00	5.00
Abnormal prothrombin (PIVKA II) (mIU/mL)	11.12–32.01	306.72	60.61	12.95
Urea (UREA) (mmol/L)	2.60–7.50	4.60	3.32	4.19
Creatinine (CREA) (µmol/L)	41.0–111.0	64.00	59.00	63.00
Blood sugar (GLU) (mmol/L)	3.90–6.10	6.76	/	5.00
Alpha-fetoprotein (AFP) (ng/mL)	0.00–7.00	>120,000.00	1749.11	2.23
Alpha-fetoprotein variant (ng/mL)	0.00–1.00	>120,000.00	355.10	<0.60
High-sensitivity hepatitis C virus Ribonucleic acid quantification (IU/mL)	<2.00E+01	5.07E+05	/	/
Hepatitis C ribonucleic acid typing (HCV RNA typing)	qPCR	HCV 2a, 6a	/	/
Hepatitis C virus antibody (S/CO)	0–1	16.32	/	/
Chitinase 3-like protein (ng/mL)	<79.00	1198.19	/	/
Carcinoembryonic antigen (CEA) (ng/mL)	<5.00	<0.50	/	1.77
Carcinoembryonic antigen 15-3 (CA15-3) (U/mL)	<15.00	/	/	6.99
Carcinoembryonic antigen 125 (CA125) (U/mL)	<35.00	/	/	9.74

**Table 3:** Comparison of the most important data in case 1 and case 2.

Term	Case 1	Case 2
Age	35	65
Past history	No special	Hypertension; Type II diabetes
Laboratory tests	HBsAb > 1000.00 mIU/mL	HCV-Ab 16.320S/CO
	HBcAb > 3.74S/CO	AFP > 120,000.00 ng/mL
	AFP CA199 no special	HCV RNA 5.07E+05*copies/mL
Treatment	Radical mastectomy (postoperative endocrine therapy (Goserelin injection + letrozole)) → Hepatectomy (Capecitabine, Envolimab, TACE)	Hepatectomy → Radical mastectomy (Docetaxel, Cyclophosphamide, Trastuzumab)

## 3. Discussion

MPMNs refers to 2 or more different primary cancers in the same patient occurring in the same or different organs or tissues. Metachronous tumors are more common than synchronous ones, and most of the MPMNs associated with female breast cancer reported in the literature are gynecological tumors^[8,9]^ and hematologic malignancies,^[10]^ making this case report particularly rare. In this report, both female patients were diagnosed as breast cancer with hepatic carcinoma, underwent more than 2 different surgeries, and the final pathology confirmed MPMN. In Tanjak’s study, breast cancer topped the list of the most common first primary cancers.^[11]^ The study also noted that the highest incidence of the first primary cancer in women was breast cancer (32.7%) and the highest incidence of the second primary cancer was lung cancer (20.8%). And the most common concomitant tumors are colon and rectal cancers.^[12]^

The pathogenesis of MPMNs is complex and not fully understood. It is associated with patient susceptibility, unhealthy lifestyle choices, autoimmune deficiencies, genetic factors, carcinogenic factors in the environment, as well as treatment methods such as radiation therapy and chemotherapy.^[13]^ While risk factors like smoking, alcohol consumption, and unhealthy lifestyle choices can potentially be eliminated, there are also uncontrollable and immutable risk factors, such as genetic predisposition, as well as the irreversible effects of cancer treatments.^[9]^ We believe that the presence of hepatitis B virus and hepatitis C virus detected in the 2 patients reported in this case played a role in the development of their liver cancer.

Due to the rarity of MPMNs, they are often confused with metastasis or recurrence of malignant tumors in clinical practice. Specifically, the clonal origin of the secondary tumor in MPMNs differs from the primary tumor, whereas it is the same in metastatic tumors.^[9]^ However, due to the homogeneity of MPMNs or exposure to the same carcinogens, mutations in genetic loci may be identical, leading to misdiagnosis of MPMNs as metastatic cancers. Similarly, due to differences in the local microenvironment, tumor cells may further alter their genetic material and biological behavior during colonization and proliferation in new metastatic sites, resulting in significant differences in phenotype compared to the primary site, which may lead to misdiagnosis of metastatic cancers as MPMNs.^[14]^

Clarifying the characteristics of each tumor lesion is crucial for the diagnosis and treatment of MPMNs patients. Clinicians need to be vigilant about such cases, not only because it is responsible for the diagnosis and treatment of patients, but also because these patients are particularly useful for extensive genome-wide discovery studies and research on rare, high-risk factors.^[15]^ In our case report, the microscopic morphology of the breast mass was completely different from that of the liver mass, suggesting that the patient’s 2 tumors may have different genetic backgrounds. However, in actual clinical work, it may affect the patient’s treatment plan due to the inaccurate results of a single biopsy due to the deviation of the sampling site or insufficient sampling volume. When a suspected diagnosis of breast cancer with liver metastasis is made, it is recommended to consider extensive resection biopsy combined with genetic sequencing techniques to enhance the accuracy of the diagnosis.

Therapeutically, there is no unified treatment plan at present. Clinically, some factors such as tumor stage and pathological type, age of patients, underlying diseases, tolerance to treatment and life expectancy need to be considered in the treatment of MPMNs.^[16]^ How can the best treatment plan be chosen in clinical practice? The most important thing in the clinical treatment of MPMNs is to determine which tumor to treat first, and how to arrange further treatment according to individual tumor risk. Previous studies have argued that the treatment of double cancers should focus on the primary cancer, and the corresponding treatment strategies should be tailored according to the characteristics of the primary tumor.^[9]^ Treatment of the first primary malignancy should be planned to ensure that subsequent malignancies are not adversely affected by increased toxicity or pharmacological interactions.^[17]^ However, at present, surgeons have little agreement on the surgical treatment order of simultaneous MPMNs in different parts. It has been reported that priority treatment is recommended for tumors that are unfavorable to the survival or quality of life of patients. The prognosis of patients undergoing surgical treatment on the basis of comprehensive treatment is better than that of simple surgical treatment.^[18]^

However, this article proposes a novel therapeutic approach that views the 2 cancers as independently occurring entities and prioritizes the management of the more severe lesion. Both patients received individualized treatment and had an uneventful recovery after multidisciplinary treatment. While this approach somewhat overlooks the potential homogeneity in pathogenesis between the 2 cancers, the rarity of homogeneity in tumors encompassed by MPMNs and the favorable survival outcomes of the 2 patients in this case report provide certain support for our treatment modality. So we strongly recommend a multidisciplinary treatment model for such patients, and design treatment strategies including chemotherapy or radiotherapy according to the current guidelines for primary tumors to provide the best treatment plan and ensure good survival results for patients. For the clinical regimes of the patients involved in this case report, it provides new intervention and treatment ideas for patients with breast cancer-associated MPMNs to improve quality of life and prognosis.

What’s more, no consensus has been established with regard to prognostic factors for MPMNs. Study have shown that clinical stage, type of surgery, and type of tumor last diagnosed are related to patient prognosis.^[18]^ Among them, age > 65 years and distant metastasis were independent poor prognostic factors for overall survival.^[19]^ However, in our case report, the patient in case 1 was younger than that in case 2, and had no special manifestations in the past history. Unfortunately, during the course of treatment, the liver lesions in case 1 recurred after surgery, which made the treatment more complicated. In other words, patients in case 2 in this report had better quality of life and prognosis, and the reasons may be related to genetic factors, the malignancy of the disease, the choice of treatment, and the psychological state of the patient. It has been reported that immunosuppression after chemotherapy may be one of the causes of second primary liver cancer in women with breast cancer.^[20]^ Fortunately, in our report, breast lesions in case 2 were treated not only with neoadjuvant chemotherapy, but with combined immunotherapy throughout the treatment cycle. Based on this, we boldly speculate that the implementation of immunotherapy may alleviate the problem of immunosuppression after chemotherapy in case 2 to a certain extent, thus making her prognosis of liver disease relatively better.

Overall, we report 2 extremely rare cases of female breast cancer with concurrent primary intrahepatic cholangiocarcinoma/mixed liver cancer. It has to be admitted that because both are diagnosed with breast cancer as the first diagnosis, liver space-occupying lesions are considered to be metastatic lesions in the discovery. Fortunately, MPMNs was confirmed by pathology after extensive surgical resection and individualized treatment was followed. Specifically, it was previously believed that the treatment of MPMNs should focus on the first primary cancer. However, we believe that the tumors contained in MPMNs are different in nature, so the tumors in MPMNS are regarded as independent, giving priority to the treatment of more serious lesions, and the patients are currently living well.

## 4. Conclusion

MPMNs exhibit high invasiveness and poor prognosis. The concurrence of breast cancer with primary liver tumors is clinically rare and prone to misdiagnosis as liver metastases, with histopathological examination being the optimal diagnostic method. In managing MPMNs, a multidisciplinary team should consider all risk factors and provide the most appropriate management plan. Early intervention aims to reduce the incidence of MPMNs at the source. Once MPMNs occur, treatment plans should be tailored to the severity of the patient’s condition, offering rational and personalized therapeutic strategies. It is noteworthy that, further research is needed to analyze clinical prognostic factors and establish a staging system for predicting outcomes given heterogeneity and bias.

## Author contributions

**Conceptualization:** Chenyan Long, Miduo Tan, Liu Luo, Yuqin Wei.

**Data curation:** Miduo Tan, Liu Luo, Taoli Wang, Zhiyong Zhang, Yuqin Wei.

**Formal analysis:** Chenyan Long.

**Investigation:** Chenyan Long, Liu Luo, Zhiyong Zhang.

**Methodology:** Miduo Tan, Liu Luo.

**Resources:** Miduo Tan, Taoli Wang, Zhiyong Zhang.

**Software:** Taoli Wang, Yuqin Wei.

**Supervision:** Chenyan Long, Miduo Tan, Zhiyong Zhang.

**Validation:** Chenyan Long, Miduo Tan, Taoli Wang, Yuqin Wei.

**Visualization:** Miduo Tan, Yuqin Wei.

**Writing – original draft:** Chenyan Long, Miduo Tan, Liu Luo, Yuqin Wei.

**Writing – review & editing:** Chenyan Long, Yuqin Wei.
